# The Nucleocapsid Protein of Human Coronavirus NL63

**DOI:** 10.1371/journal.pone.0117833

**Published:** 2015-02-20

**Authors:** Kaja Zuwała, Anna Golda, Wojciech Kabala, Michał Burmistrz, Michal Zdzalik, Paulina Nowak, Sylwia Kedracka-Krok, Mirosław Zarebski, Jerzy Dobrucki, Dominik Florek, Sławomir Zeglen, Jacek Wojarski, Jan Potempa, Grzegorz Dubin, Krzysztof Pyrc

**Affiliations:** 1 Microbiology Department, Faculty of Biochemistry, Biophysics and Biotechnology, Jagiellonian University, Gronostajowa 7, 30–387, Krakow, Poland; 2 Malopolska Centre of Biotechnology, Jagiellonian University, Gronostajowa 7, 30–387, Krakow, Poland; 3 Department of Physical Biochemistry, Faculty of Biochemistry, Biophysics and Biotechnology, Jagiellonian University, Gronostajowa 7, 30–387, Krakow, Poland; 4 Division of Cell Biophysics, Faculty of Biochemistry, Biophysics and Biotechnology, Jagiellonian University, Krakow, Poland; 5 Department of Cardiac Surgery and Transplantology, Silesian Center for Heart Diseases, Szpitalna 2, 41–800, Zabrze, Poland; 6 Oral Health and Systemic Disease Research Group, School of Dentistry, University of Louisville, Louisville, KY, United States of America; Institut National de la Santé et de la Recherche Médicale, FRANCE

## Abstract

Human coronavirus (HCoV) NL63 was first described in 2004 and is associated with respiratory tract disease of varying severity. At the genetic and structural level, HCoV-NL63 is similar to other members of the *Coronavirinae* subfamily, especially human coronavirus 229E (HCoV-229E). Detailed analysis, however, reveals several unique features of the pathogen. The coronaviral nucleocapsid protein is abundantly present in infected cells. It is a multi-domain, multi-functional protein important for viral replication and a number of cellular processes. The aim of the present study was to characterize the HCoV-NL63 nucleocapsid protein. Biochemical analyses revealed that the protein shares characteristics with homologous proteins encoded in other coronaviral genomes, with the N-terminal domain responsible for nucleic acid binding and the C-terminal domain involved in protein oligomerization. Surprisingly, analysis of the subcellular localization of the N protein of HCoV-NL63 revealed that, differently than homologous proteins from other coronaviral species except for SARS-CoV, it is not present in the nucleus of infected or transfected cells. Furthermore, no significant alteration in cell cycle progression in cells expressing the protein was observed. This is in stark contrast with results obtained for other coronaviruses, except for the SARS-CoV.

## Introduction

Coronaviruses cause a variety of diseases in animals, whereas human infections are almost exclusively associated with respiratory tract infections (RTI). Contemporary taxonomy divides the *Coronavirinae* subfamily into four genera (alpha, beta, gamma, and delta). Only the alpha and beta species infect humans [1]; these include two species identified in the 1960s (human coronavirus (HCoV) 229E and HCoV-OC43) [2–5], and four species identified within last 10 years: severe acute respiratory syndrome coronavirus (SARS-CoV) [6–8], HCoV-NL63 [9,10], HCoV-HKU1[11], and Middle East respiratory syndrome coronavirus (MERS-CoV) [12].

HCoV-NL63 was first described in 2004 in a clinical sample from a child suffering from a respiratory condition that tested negative for all known respiratory pathogens [9,13]. Subsequent studies demonstrated that infection with the virus is generally associated with upper and lower RTIs of varying severity, although the disease is usually self-limiting and causes only common cold-like symptoms [14–18]. However, fatal cases have also been reported [19,20]. In addition, HCoV-NL63 is the major etiological factor of croup in young children [14,21,22]. The overall incidence of the virus in patients suffering from RTIs is estimated to be 2–10%, and is highest in winter and spring [14–18].

At the genetic level, HCoV-NL63 is similar to other members of the *Coronavirinae* subfamily [9]. Detailed analysis, however, reveals several unique features. For example, instead of aminopeptidase N (CD13), which is used by other members of the alphacoronaviruses genus, HCoV-NL63 uses angiotensin converting enzyme 2 (ACE2) as its cellular receptor [23–27]. This receptor specificity is shared with the highly virulent SARS-CoV, which raises questions regarding virulence determinants and makes this coronavirus an interesting study subject.

The structure of the large HCoV-NL63 genomic RNA molecule is similar to that of other members of the family, and encodes the viral replicative machinery within the 5’ part and the structural proteins within the 3’ part [9,28,29]. It is worth noting that some of the structural proteins are also important for replication; among these, the nucleocapsid (N) protein is one of the most intriguing. This multi-functional protein is the major coronaviral protein produced in infected cells [28,30]. The protein forms a ribonucleoprotein together with genomic RNA, which is then inserted into a lipid envelope carrying other structural proteins that are responsible for membrane curvature formation, vesicle scission, and interaction with cellular receptors [31]. The RNA-binding ability of coronaviral N protein is important not only for genome encapsidation, but also for discontinuous transcription and polymerase template switching [32,33]. Furthermore, the protein may also modulate cellular physiology, thereby transforming the cell into a robust virus production plant. The N protein of some coronaviral species can affect cell cycle progression, cytoskeleton organization, gene transcription, and apoptosis induction in infected cells [34–38]. Furthermore, the protein enables the virus to avoid detection by pathogen pattern recognition molecules, including Mda5 and RIG-I helicases [39]. This list of functions may not yet be complete, although its current versatility highlights the importance of the N protein.

The aim of the present study was to characterize the HCoV-NL63 N protein (referred to hereafter as NL63-N). The results clearly show that NL63-N occupies a rather unique cellular localization as it is not translocated to the nucleus in any of the cell lines or primary cells examined. Consistently, we did not observe any marked alteration in cell cycle progression in cells expressing the NL63-N. Biochemical analyses revealed that the NL63-N shares characteristics with homologous proteins encoded by the genomes of other coronaviruses. It forms oligomers via its C-terminal domain (CTD) and binds nucleic acids *via* its N-terminal domain (NTD). Notably, the complete NL63-N protein was rather unstable, whereas the CTD showed exquisite stability.

## Materials and Methods

### *In silico* analysis

Multiple sequence alignments were prepared with ClustalX 2.0 (normal sequence alignment) and manually edited in BioEdit ver. 7.1.3.0. Analysis of the protein sequence for nuclear localization signals was carried out with PSORT II server (http://psort.hgc.jp/) [40,41].

### Cell culture

LLC-MK2 cells (ATCC: CCL-7; *Macaca mulatta* kidney epithelial cell line) were maintained in minimal essential medium (MEM), containing 2 parts of Hank’s MEM and 1 part of Earle’s MEM (PAA Laboratories, Austria) supplemented with 3% heat-inactivated fetal bovine serum (FBS) (PAA Laboratories, Austria), penicillin (100 U/ml), and streptomycin (100 μg/ml). Cells were cultured on T75 flasks (TPP, Switzerland) at 37°C with 5% CO_2_.

293T cells (ECACC: 12022001; Human embryonic kidney SV40 transformed, genetically modified) were maintained in Dulbecco-modified Eagle’s medium (DMEM; PAA Laboratories, Austria) supplemented with 3% heat-inactivated fetal bovine serum (FBS; PAA Laboratories, Austria), penicillin (100 U/ml), and streptomycin (100 μg/ml). Cells were cultured on T75 flasks (TPP, Switzerland) at 37°C with 5% CO_2_.

293T cells (ATCC CRL-3216) were transfected with the pLKO.1.-TRC-ACE2 plasmid using polyethylenimine (PEI; Sigma-Aldrich, Poland). The plasmid was based on the Addgene plasmid 10878 [42]. At 24 h post-transfection, the cells were washed with sterile 1 × PBS and cultured at 37°C for 48 h in media supplemented with puromycin (2 μg ml^-1^) at 37°C with 5% CO_2_. Following selection, cells were passaged and the surviving clones were collected and analyzed. ACE2-expressing (ACE2^+^) cells were maintained in Dulbecco’s MEM (PAA Laboratories, Austria) supplemented with 10% FBS, penicillin (100 U ml^-1^), streptomycin (100 μg ml^-1^), ciprofloxacin (5 μg ml^-1^) and puromycin (1 μg ml^-1^). 293T_ACE2^+^ cells were maintained as wild type cells.

Human tracheobronchial epithelial cells were obtained from airway specimens resected from patients undergoing surgery under Silesian Center for Heart Diseases approved protocols. This study was approved by the bioethical committee of the Medical University of Silesia in Katowice, Poland (approval no: KNW/0022/KB1/17/10 dated on 16.02.2010). A written informed consent was obtained from all patients (2 adult patients). Primary cells detached from human bronchi and trachea with pronase E were expanded on collagen-coated (collagen type IV, Sigma-Aldrich) plastic in bronchial epithelial growth media (BEGM) to generate passage 1 cells and plated at density of 3×10^5^ cells per well on permeable Transwell supports (6.5-mm-diameter; Corning Transwell-Clear) in BEGM. Cells were cultured at 37°C in presence of 5% CO_2_ until confluence. Human airway epithelium (HAE) cultures were generated by changing the media to Air Liquid Interface media (ALI) and provision of an air-liquid interface for 6 to 8 weeks to form well-differentiated, polarized cultures that resemble the *in vivo* structure of pseudostratified mucociliary epithelium. All procedures were performed as previously described [43].

All cell cultures were routinely screened for *Mycoplasma spp*. contamination using Hoechst 33258 staining.

### Viruses

HCoV-NL63 (Amsterdam I strain) stock was generated by infecting LLC-MK2 cells. Infected cells were lysed 6 days post-infection by two freeze-thaw cycles. The virus-containing fluid was cleared by centrifugation, aliquoted and stored at -80°C. A control from mock infected cells was prepared in the same manner as the virus stocks. Virus yield was assessed by titration on fully confluent LLC-MK2 cells, according to Reed and Muench formula [44]. Cells on 96-well plates were incubated at 32°C for 6 days and the cytopathic effect was scored using an inverted microscope. All experimental procedures were conducted as previously described [45]. Virus identity was confirmed by cDNA sequencing.

### Nucleic acids

RNA from viral and control cultures was extracted using GeneJet RNA purification kit (Thermo Scientific, Lithuania), according to manufacturer’s protocol. Isolated RNA was stored at -80°C. DNA fragments were synthesized by a third party (Genomed, Poland).

### Cloning

Isolated viral RNA was reverse transcribed using High Capacity cDNA kit (Life Technologies, Poland) and used as a template for subsequent amplification.

In order to obtain eukaryotic expression vector, NL63-N gene was amplified using primers N_NL63_5HindIII (5’- GTA CAA GCT TGC CAC CAT GGC TAG TGT AAA TTG GGC C-3’) and N_NL63_3BamHI (5’- GAC TGG ATC CGC ATG CAA AAC CTC GTT GAC AAT-3’) with Marathon DNA polymerase (A&A Biotechnology, Poland). Resulting PCR product was subsequently gel purified using GeneJET Gel Extraction kit (Thermo Scientific, Lithuania) and digested with HindIII and BamHI restriction enzymes. Resulting fragment was cloned into the pmaxFP-Green-N plasmid (Lonza, Switzerland) using corresponding restriction sites. Plasmids (pmaxFP-Green-N/NL63-N) were recovered in DH5α *Escherichia coli* (Life Technologies, Poland) and their identity and sequence were confirmed by DNA sequencing (Genomed, Poland).

Expression plasmids for prokaryotic expression of the NL63-N protein were prepared using pmaxFP-Green-N/NL63-N as a template. Briefly, the fragments of N gene were amplified using primers given in parentheses: procNL63-N (5’- ATG CCC ATG GGC CAT CAC CAT CAT CAC CAC TCT GGC GAC GAC GAC GAC AAG GCT AGT GTA AAT TGG GCC GAT G-3’ and 5’- ATG CCT CGA GTT AAT GCA AAA CCT CGT TGA CAA T-3’), procNL63-20/144-N (5’- CAT AGG ATC CAG AAA ACC TGT ATT TTC AGG GAT CAT TTT ACA TGC CTC TTT TG-3’ and 5’- CAG CAA GCT TTT AAG AGC GAT CCT CAA ACT CAA C-3’) and procNL63-221/340-N (5’- CAT AGG ATC CAG AAA ACC TGT ATT TTC AGG GAT CTC AAC CCA GGG CTG ATA AG-3’ and 5’- CAG CAA GCT TTT ATG ACT GCA TTT CTT TGA TAG-3’) and cloned into the pET Duet 1 plasmid (Clonetech, USA). The element encoding 6 × His tag was introduced at the N-terminus of the gene. Three plasmids for prokaryotic expression were generated: procNL63-N, procNL63-20/144-N (for expression of the N terminal—domain), and procNL63-221/340-N for expression of the C-terminal domain.

### Transfection of eukaryotic cells

Plasmid pmaxFP-Green-N/NL63-N or control plasmids were transfected to 293T cells using cationic carrier (polyethylenimine, PEI; Sigma-Aldrich, Poland). Briefly, 2 × 10^5^ cells were seeded onto collagen-coated (Purecol; Advanced Biomatrix, USA) glass coverslips in a 6-well plate. Next day media was removed, cells were washed with 1 × PBS and overlaid with 2 ml of DMEM supplemented with 4 μg of PEI and 4 μg of plasmid. 24 h post-transfection coverslips were harvested for analysis.

In order to test subcellular localization of the N protein in LLC-MK2 cells, the maxFP-Green-N/NL63-N encoding RNA was prepared based on the original plasmid. Briefly, the plasmid was used as a template with primers SP6_NEGFPmRNA (5’- ACT GAC TGA TTT AGG TGA CAC TAT AGA AGN GAA GCT TGC CAC CAT GGC TAG TG -3’) and EGFPmRNA_R (5’- TTT TTT TTT TTT TTT TTT TTT CAT TAA TGC AAA ACC TCG TTG AC -3’), where the 5’ primer carries the SP6 promoter. *In vitro* transcription was carried out using the mMessage mMachine SP6 kit (Life Technologies, Poland). Further, RNA was polyadenylated using Poly(A) Tailing Kit (Life Technologies, Poland) and transfected into cells using TransIT-mRNA Transfection Kit (Mirus, USA), as advised by the manufacturer.

RNA encoding maxFP-Green protein (control) was prepared and transfected in the same manner using primers SP6_GFPmRNA (atg cAT TTA GGT GAC ACT ATA GAT GGA GAG CGA CGA GAG CGG CCT GC) and GFPmRNA_R (TTT TTT TTT TTT TTT TTT TTT TTT TTT TTT TTT TTT TTT TTC ATT ATT CTT CAC CGG CAT CTG CAT C).

In order to assess the influence of the N protein on cell cycle, the N-encoding RNA was prepared based on the original plasmid and transfected in the same manner as described above. Primers SP6_NmRNA (5’- TCG GCC TCG TAG GCC ATT TAG GTG ACA CTA TAG AAG NCT GAG AGA ACC CAC TGC TTA C -3’) and NmRNA_R (5’- TTT TTT TTT TTT TTT TTT TTT CAT TAA TGC AAA ACC TCG TTG AC -3’) were used, where the 5’ primer carries the SP6 promoter.

### Cell cycle assessment

Cells transfected with mRNA encoding the N protein or control cells were harvested 48 h post-transfection by trypsinization and pelleted in sterile 1 × PBS. After fixation in 70% EtOH for 2 h on ice, cells were incubated in staining solution (50 μg/ml propidium iodide and 10 μg/ml RNase A in sterile 1 × PBS; Sigma–Aldrich, Poland) for 30 min at 37°C. The N protein was visualized with monoclonal antibody specific to NL63-N (Ingenasa, Spain) and secondary Alexa Fluor 488 goat anti-mouse antibody (Life Technologies, Poland). Cells expressing the N-NL63 protein were analyzed by flow cytometry (FACSCalibur, Becton Dickinson) as previously described [46].

Cells treated with nocodazole (Sigma–Aldrich, Poland), a mitotic spindle poison, were sampled after 24 h and evaluated as positive control for cell cycle arrest. Obtained data were analyzed using ModFit LT software (Verity Software House, USA). All experiments were conducted independently at least three times.

### Fluorescent microscopy

Cells were fixed using 4% formaldehyde solution in sterile 1 × PBS for 15 minutes. Subsequently, cells were washed three times with 1 × PBS and incubated with 0.1% Triton solution in 1 × PBS to remove lipid fraction. Further, cells were incubated in blocking buffer (10% of BSA; BioShop, Canada, 0.1% Tween 20; BioShop, Canada in 1 × PBS) for 60 minutes.

For detection of HCoV-NL63 N protein mouse monoclonal anti-HCoV-NL63-N antibody (diluted 4000 ×, Ingenasa, Spain) was incubated with the sample for 1 hour at 4°C, followed by incubation with an anti-mouse Alexa Fluor 488 (dilution 400 ×, Thermo Fisher Scientific, Poland) for 1 hour at 4°C. For visualization of nucleic acids, DAPI dye (1 μg/ml; Sigma-Aldrich, Poland) was used. Fluorescent images were acquired with Leica TCS SP5 II confocal microscope (Leica Microsystems GmbH, Germany). Images were pre-processed using Leica Application Suite Advanced Fluorescence LAS AF v. 2.2.1 (Leica Microsystems GmbH) and further deconvolved with Huygens Essential package ver. 4.4 (Scientific Volume Imaging B.V., the Netherlands). All experiments were conducted independently at least three times.

### Prokaryotic expression and purification of the N protein

The NL63-N NTD (amino acids 2–144) and CTD (amino acids 221–340) expression constructs were designed *in silico* by analysis of sequence alignments, comparative modeling and literature data. The sequences and structures of the homologous coronavirus nucleocapsid polypeptides used are listed in **Table 1**. The strategy is described in the Supporting Information section. Sequence sets were prepared using BLAST and SPDBV. The comparative modeling was performed with SPDBV, Coot and PyMOL [47–49]. Procedure of gene amplification and plasmid preparation is described above.

**Table 1:** References to sequence and structural data on polypeptides used to design the expression constructs of HCoV-NL63 nucleocapsid N- and C- terminal domains.

PDB ID	Uniprot ID	Source	Polyprotein Description	Reference
2bxx	P69596	Avian IBV (strain Beaudette)	Nucleocapsid NTD	[50]
2c86	P69598	Avian IBV (strain Beaudette US)	Nucleocapsid NTD	[56]
2gec	P32923	Avian IBV (strain Grey)	Nucleocapsid NTD	[56]
3hd4	P03416	MHV	Nucleocapsid NTD	[80]
2ofz	P59595	Human SARS CoV	Nucleocapsid NTD	[51]
2og3	P59595	Human SARS CoV	Nucleocapsid NTD	[51]
2ca1	P69596	Avian IBV (strain Beaudette)	Nucleocapsid CTD	[56]
2ge7	P32923	Avian IBV (strain Grey)	Nucleocapsid CTD	[56]
2ge8	P32923	Avian IBV (strain Grey)	Nucleocapsid CTD	[56]
2cjr	P59595	Human SARS CoV	Nucleocapsid CTD	[81]
2jw8	P59595	Human SARS CoV	Nucleocapsid CTD	[82]

In order to express the N protein, NTD and CTD in *E*. *coli*, respective plasmids were transformed to BL21 cells and further cultured in LB media supplemented with ampicillin (100 μg/ml) at 37°C, until the optical density (λ = 600nm) reached 0.5–0.6. Expression was induced by addition of IPTG (1 mM) and continued overnight at 20°C. Subsequently cells were pelleted by centrifugation and suspended in lysis buffer (50 mM Tris, 500 mM NaCl, 20 mM Imidazol pH 8.0). For the full length protein, the buffer was supplemented with 5 mM β-mercaptoethanol. Bacterial cells were lyzed by sonication, and cellular debris was removed by centrifugation. Proteins of interest were recovered by affinity chromatography (Ni Sepharose 6 Fast Flow, GE Healthcare, Poland), ion-exchange chromatography (Resource Q, GE Healthcare, Poland) and size exclusion chromatography (Superdex S75, GE Healthcare, Poland). Protein was detected using Western-blotting technique and anti-6 × His antibodies (Life Technologies, Poland).

### Mass spectrometry

Samples for mass spectrometry were prepared by dialysis into 50 mM NH_4_HCO_3_, pH 7.8. Measurements were performed using the MicroTOF-QII mass spectrometer (Bruker, Germany) in positive ionization mode, using Appollo Source ESI sprayer. Prior to measurements the device was calibrated with TuneMix solution. The obtained MS spectra were analyzed using Data Analysis 4.0 software (Bruker, Germany). Molecular weight of proteins was confirmed using Maximum Entropy algorithm for MS spectra deconvolution (Bruker, Germany).

### Electron microscopy

Protein preparations were overlaid on the poly-L-lysine coated glass slides (diameter of 16 mm). Samples were fixed with 2.5% glutaraldehyde in 0.1M sodium cacodylate buffer pH = 7.4 for 20 minutes. Subsequently, samples were washed with the abovementioned buffer and gently dehydrated by using solutions of ethanol in a graded series of concentrations.

Preparations were dried in a critical point dryer (Quorum Technologies, United Kingdom). Slides were mounted on holders using self-adhesive carbon discs (TAAB laboratories, United Kingdom) and sputter coated with gold (ion sputter JFC-1100E; JEOL, Japan). Electron micrographs were prepared using scanning electron microscope JSM-5410 (JEOL, Japan).

### Differential Scanning Calorimetry (DSC)

DSC experiments were performed on a Calorimetry Sciences Corporation 6100 Nano II differential scanning calorimeter with a cell volume of 0.3228 ml. The heat capacity of 1 mg/ml protein solution in 50 mM NH_4_HCO_3_, pH 7.8 was recorded relative to pure buffer at a scan rate of 1 K/min. T_m_ was defined as the temperature corresponding to the peak maximum. The enthalpy was calculated by integration of the area under transition peak.

DSC technique allows to check the validity of the two-state model by calculation of van’t Hoff enthalpy (*ΔH*_*vH*_):

$$\Delta H_{vH}=4R\cdot T_{m}^{2}\cdot \frac{C_{p}^{exc,\max}}{\Delta H\left(T_{m}\right)}$$

where $C_{p}^{exc,\max}$ is heat capacity at the *T*_*m*_ measured with respect to the chemical baseline. The ratio $C_{p}^{exc,\max}∕\Delta H\left(T_{m}\right)$ is sensitive to the shape (width) of the transition. If two-state model holds true, the van’t Hoff and calorimetric enthalpies are equal within the experimental uncertainty, and so the *ration = ΔH(T_m_)/ΔH_vH_* should be equal to unity.

### Protein electrophoresis and EMSA

Protein electrophoresis in denaturing conditions was carried out in Schagger & von Jagow system. Electrophoretic separation was carried out at 75 V (stacking) / 135 V (separation). Proteins were visualized using Coomassie Brilliant Blue G-250 (Serva, Germany). Page Ruler Plus (Thermo Scientific, Lithuania) was used as a prestained protein size marker.

For EMSA assay 10 μg of RNA or DNA corresponding in sequence to the N-NL63 gene (prepared in the same manner as for the transfection of eukaryotic cells) was incubated in buffered solution (5 mM Tris, 50 mM NaCl, pH8.0) with 10 μg of the NTD or CTD for 30 minutes at room temperature. Subsequently, samples were separated on agarose gels and signal from nucleic acids was visualized with ethidium bromide staining. All experiments were conducted independently at least three times.

### Chemical cross-linking assay

In order to assess whether the NTD or CTD are able to form oligomers, 50 μg of the protein was mixed with glutaraldehyde (0.007%; Serva). Following 15 minute incubation at room temperature 0.5 μl of 1M Tris solution was added to the mixture, samples were mixed with protein sample buffer, denatured at 95°C and loaded onto the polyacrylamide gel. All experiments were conducted independently at least three times.

### Nucleotide sequence accession numbers

The sequences of DNA, RNA and proteins used within the study correspond to those of HCoV-NL63 isolate Amsterdam 1 (GenBank accession number: NC_005831). Accession numbers for N proteins of different coronaviruses are provided in **S1 File**.

## Results

### *In silico* analysis of NL63-N

NL63-N is a basic protein (predicted pI, 9.78) comprising 377 amino acids (aa). The predicted molecular weight is 42,252.47 Da. Literature data indicate that the full length coronaviral nucleocapsid protein consists of two folded domains linked by an unstructured region. In more details the N protein includes following elements: N-tail, N-terminal domain (NTD), R.S.A.G. rich linker, C-terminal domain (CTD) and C-tail [50]. The constructs of NTD and CTD used in this study were designed based on literature data, HCoV-NL63 N protein amino acid sequence alignment with known homologs and on the comparative analysis of currently available crystal structures of these homologs.

According to Saikatendu *et al*. the NTD of HCoV-NL63 N encompasses residues 17–141 [51]. Our sequence alignment and structural analysis suggests that NL63-N 2–144 fragment better reflects the full N-terminal domain. NL63-N fragment encompassing its CTD was chosen exclusively on the basis of sequence alignment and structural analysis which suggests that fragment 221–340 contains full, structurally stable CTD. The sequences and structures of N proteins used in above analysis are listed in Table 1. The analysis strategy is summarized in **S1 File** and the amino acid sequences of the final constructs of CTD and NTD are presented in **S2 File**.

*In silico* analysis conducted using PSORT II revealed that two nuclear localization signals (NLS) are buried within NL63-N: pat4 (aa 232-KKPR-235) and pat7 (aa 234-PRWKRVP-240). No bipartite NLS were detected.

### Basic properties of the N protein

**Fig 1:**
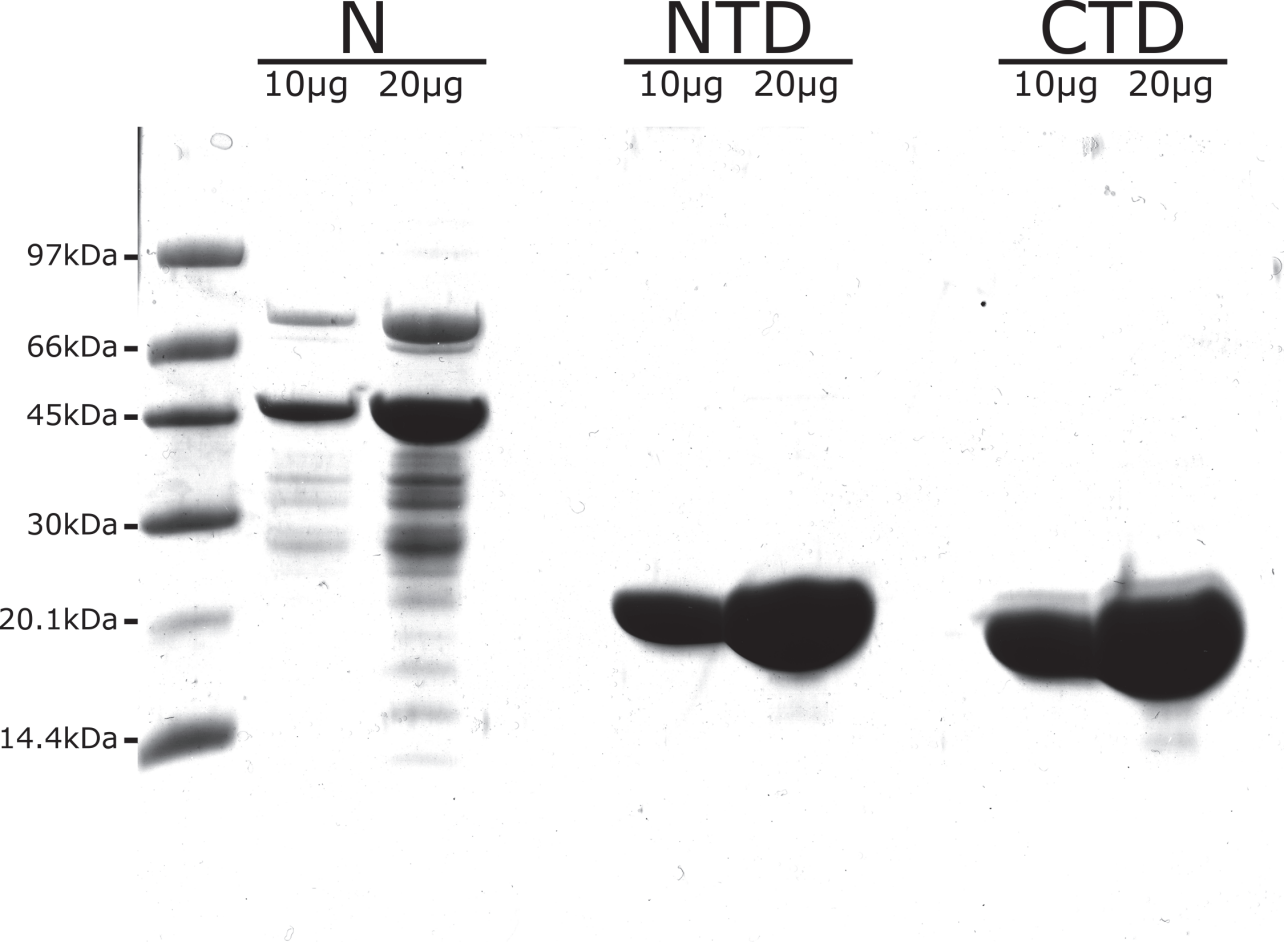
Purified N protein of HCoV-NL63 and its domains. NL63-N protein was expressed in *E*. *coli* and purified as described in the Materials and Methods section. Purity of the protein was evaluated using SDS-PAGE analysis (Coomassie brilliant blue staining). N: complete NL63-N protein, NTD and CTD: N-terminal and C-terminal domains of the NL63-N protein, respectively. For each sample two different protein quantities were analyzed (10 μg and 20 μg). LMW Amersham GE Healthcare size marker was used, and corresponding sizes are presented on the left side of the figure.

The complete NL63-N protein and its CTD and NTD were expressed in *E*. *coli* BL21 cells. NTD and CTD were purified to homogeneity whereas the full length N-protein was purified to about 80% homogeneity as demonstrated by SDS-PAGE (**Fig. 1**). The identity of purified proteins was confirmed with mass spectrometry (data not shown).

**Fig 2:**
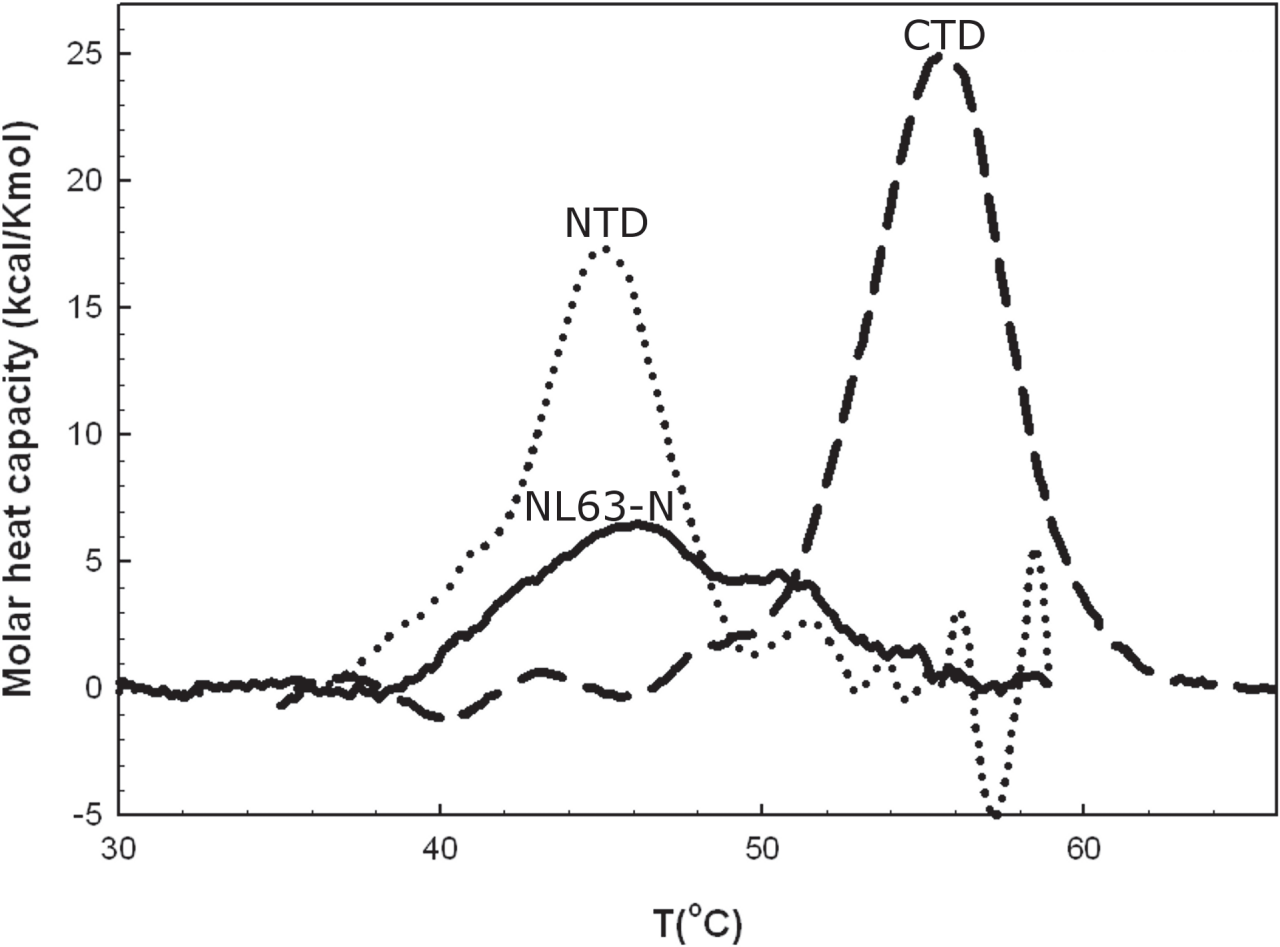
DSC curves for the first heating scans for the complete NL63-N protein and its domains. NL63-N: complete NL63-N protein, NTD: N-terminal domain, CTD: C-terminal domain. Samples were suspended in 50 mM NH_4_HCO_3_, pH 7.8 and scanned at the rate of 1 K/min. Protein concentration was 1 mg/ml.

We next used differential scanning calorimetry (DSC) to examine thermal stability of the proteins. The DSC curves for the first heating scans obtained for NL63-N, the CTD, and the NTD are shown in **Fig. 2**. NL63-N underwent irreversible denaturation and showed a broad transition curve. The denaturation temperature was estimated at 45.7°C, with an enthalpy change of approximately 80 kcal/mol (**Table 2**). However, due to low signal to noise ratio the resulting baseline was variable and these values can only be treated as rough estimates. Nevertheless, the low enthalpy value suggests that NL63-N protein is relatively unstable. Unfolding of the NTD was irreversible and accompanied by protein aggregation (indicated by the exotherm present in the high temperature region of the DSC curve). The transition temperature was 45°C and the ΔH_cal_ was 104.4 kcal/mol. Surprisingly, thermal transition of the CTD was fully reversible, showing T_t_ of 55.7°C, a ΔH_cal_ of 143.6°kcal/mol, and a ΔS_cal_ of 0.44 kcal/Kmol. Thermal transition of the CTD was cooperative, with a van't Hoff enthalpy/calorimetric enthalpy ratio (ΔH_van’Hoff_/ ΔH_cal_) of 1.03.

**Table 2:** Thermodynamic parameters describing N protein and its domains.

	WT	CTD	NTD
ΔH kcal/mol	81.05	143.6	104.4
**Tt (°C)**	45.7	55.7	45.0
ΔS kcal/K*mol	-	0.44	-

### Oligomerization of the N protein is mediated by the CTD

The coronaviral nucleocapsid forms a protective scaffold around the viral RNA [52–54]. The N protein forms oligomers *via* specific interactions between different regions within the protein [55–57]. To confirm this assumption for HCoV-NL63, we performed mass spectrometry analyses. The results confirmed the presence of complete N protein dimers. Furthermore, similar results were obtained for the CTD but not NTD, suggesting that CTD harbors the sites responsible for N protein dimerization (**Table 3**).

**Table 3:** N protein and CTD are able to form dimers.

	Measured MW [Da]	
Proteins	monomer	dimer
WT	43 733.78	87 466.42
NTD	16 841.73	-
CTD	15 934.36	31 868.64

Molecular weight values for WT nucleocapsid protein and its domains, as determined using mass spectrometry. Experimental details are provided in the text.

**Fig 3:**
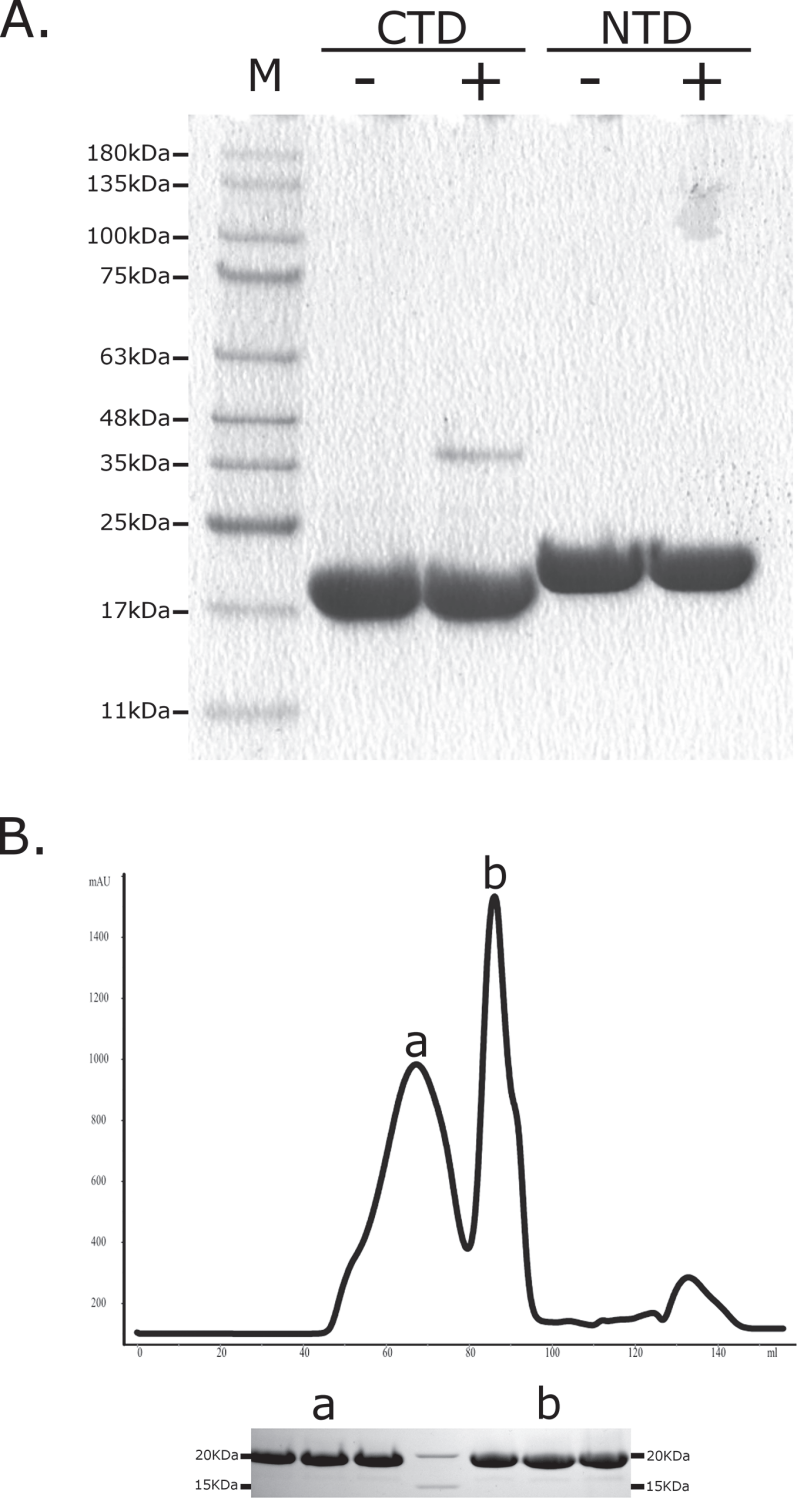
Oligomerization of the CTD of the NL63-N protein. (**A**.) Putative CTD and NTD complexes were stabilized by means of protein crosslinking and subsequently analyzed with SDS-PAGE electrophoresis (Coomassie brilliant blue staining). HMW Jena Bioscience GmbH size marker was used, and corresponding sizes are presented on the left side of the figure. “+” and “-” marks denote samples treated with glutaraldehyde or control samples, respectively (**B**.) The CTD was analyzed with size exclusion chromatography; two peaks visible on the chromatogram (a and b) represent dimeric and monomeric forms of the CTD respectively, as inferred form retention time and SDS-PAGE analysis (shown at the bottom of the figure; three lanes presented for each peak represent three independent fractions).

The mass spectrometry results were confirmed by protein crosslinking studies. Incubating the CTD in the presence of glutaraldehyde followed by SDS-PAGE analysis revealed the presence of protein dimers and higher molecular weight oligomers (**Fig. 3A**). Similar results were obtained using size exclusion chromatography, showing that ~40% of the protein is present as dimers (**Fig. 3B**). Dimerization was not observed for NTD.

### Nucleic acid binding

**Fig 4:**
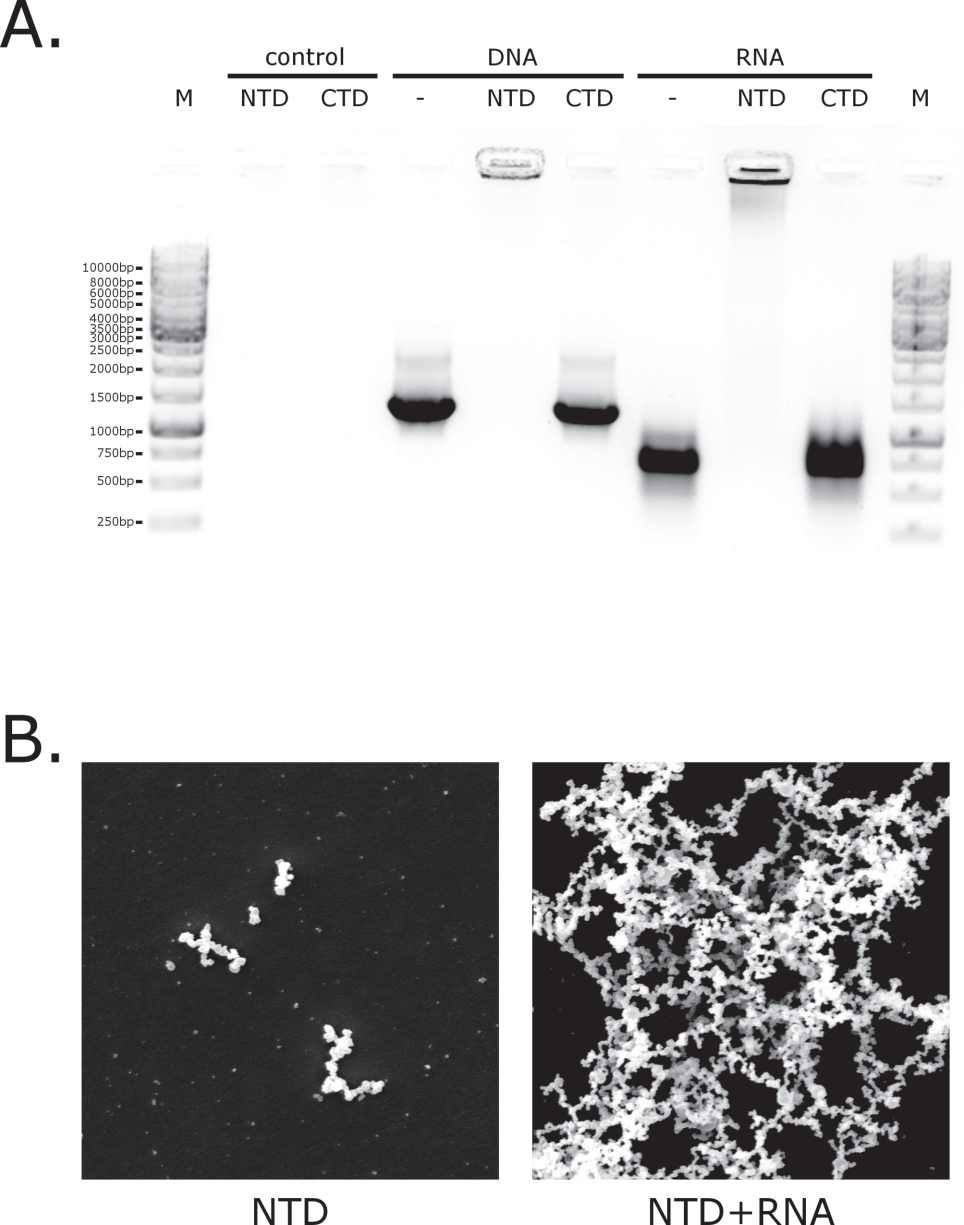
Interaction of the CTD and NTD with RNA and DNA. **(A)** RNA or DNA samples were pre-incubated with the protein and subsequently separated on the agarose gel. Shifts (the nucleoprotein complex does not leave the well in this case) observed in the lines containing RNA and DNA pre-incubated with the NTD suggest strong RNA-NTD and DNA-NTD interaction. **(B)** Electron microscopy images of the NTD in the absence and in the presence of RNA. Micrographs were prepared using scanning electron microscope JSM-5410.

We next performed an electrophoretic mobility shift assay to determine whether the N protein interacts with nucleic acids. Briefly, samples containing nucleic acids were separated in agarose gel under native conditions in the presence/absence of the CTD or the NTD. Nucleic acids were detected by ethidium bromide staining. As shown in **Fig. 4**, the NTD binds nucleic acids (both DNA and RNA), as demonstrated by retarded RNA and DNA migration. The CTD did not bind nucleic acids. We also conducted similar analysis for the complete N protein (data not shown). Obtained results suggested that the complete N protein has lower nucleic acid binding ability or is more specific compared to the NTD. However, due to rapid degradation of the complete N protein into separate domains and resulting presence of the free NTD in the solution these results were inconclusive.

### Subcellular localization of the N protein

Coronaviral N proteins localize to the cytoplasm, where they are involved in virus replication and assembly. However, the N proteins of almost all coronaviruses (except for SARS-CoV) also localize to the nucleus or to micronuclei [58–61].

**Fig 5:**
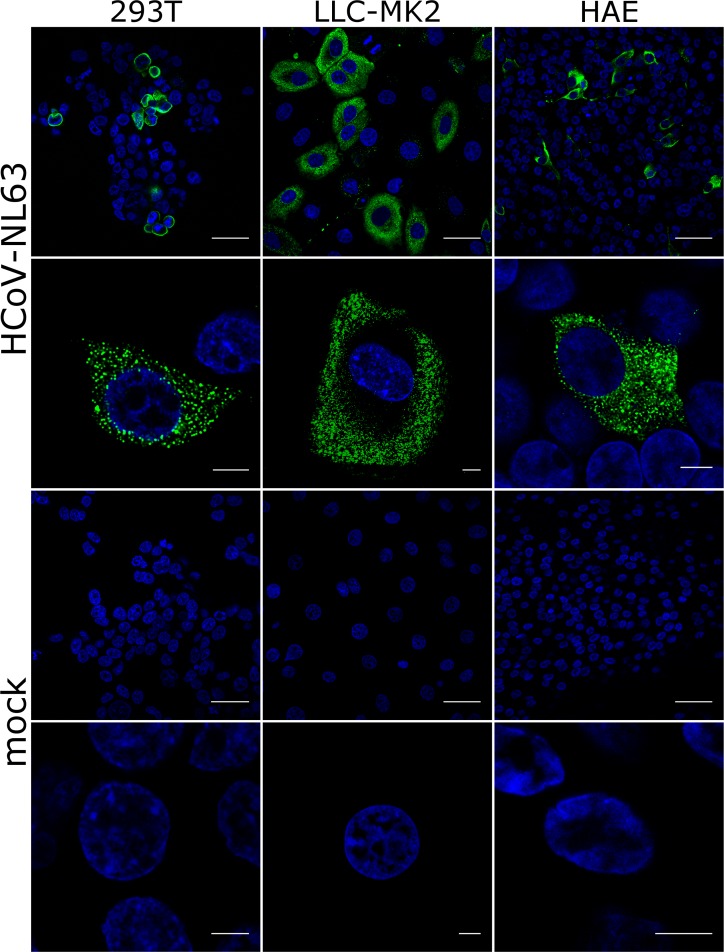
Localization of the N protein in cells infected with HCoV-NL63. Three culture systems were used: 293T_ACE2^+^ cells, LLC-MK2 cells and fully differentiated human airway epithelial cultures. Single confocal planes are presented. Blue color denotes nuclei, while green represents localization of the HCoV-NL63 nucleocapsid protein. Top image in each set: scale bar corresponds to 40 μm; bottom image: scale bar corresponds to 5 μm.

**Fig 6:**
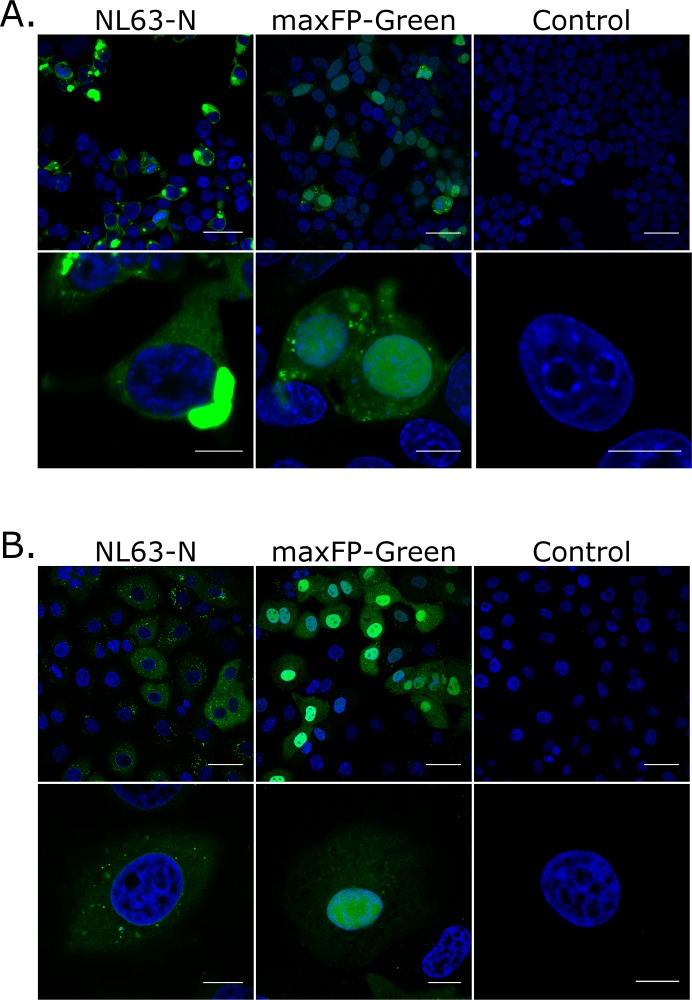
Localization of the N protein fused with the maxFP-Green protein in eukaryotic cells. 293T_ACE2^+^ (**A**.) and LLC-MK2 (**B**.) cells were used. Single confocal planes are presented. Blue color denotes nuclei, while green represents localization of the HCoV-NL63 nucleocapsid protein. Top image in each set: scale bar corresponds to 40 μm; bottom image: scale bar corresponds to 10 μm. NL63-N: cells expressing N protein fused with the maxFP-Green protein; maxFP-Green: cells expressing maxFP-Green protein; Control: mock-transfected cells.

To examine the subcellular localization of the NL63-N, cultures of 293T_ACE2^+^, LLC-MK2, and HAE cultures were infected with the HCoV-NL63 virus. Subsequently, the cells were fixed and stained with antibodies specific for the N protein. In all cell types tested the protein localized exclusively in the cytoplasm (**Fig. 5, S3, S4, S5 Files**). To test whether the observed lack of nuclear localization of NL63-N does not result from insufficient nuclear staining, 293T cells were also transfected with pmaxFP-Green-N/NL63-N plasmid. LLC-MK2 cells were transfected with maxFP-Green/NL63-N encoding mRNA due to poor transfection efficiency using conventional DNA delivery methods. MaxFP-Green protein was used as a control. We then examined the subcellular localization of NL63-N using confocal microscopy (**Fig. 6A** and **6B**). maxFP-Green-labeled NL63-N localized exclusively to the cytoplasm in all tested cell types and no staining was observed in the nucleus. Overexpression of the N protein resulted in formation of large deposits of the N protein in the cytoplasm, which may be attributed to vast overexpression of the protein, as infection of these cells did not result in formation of such structures. Similar patterns were previously seen for other coronaviruses [62].

### Role of NL63-N in cell cycle progression

Previous reports show that expression of the coronaviral N protein results in delayed cellular growth. These observations were supported by biochemical studies showing that the N protein may actively participate in cell cycle regulation in infected cells by localizing to the nucleus and interacting with cyclins [36–38,61].

**Fig 7:**
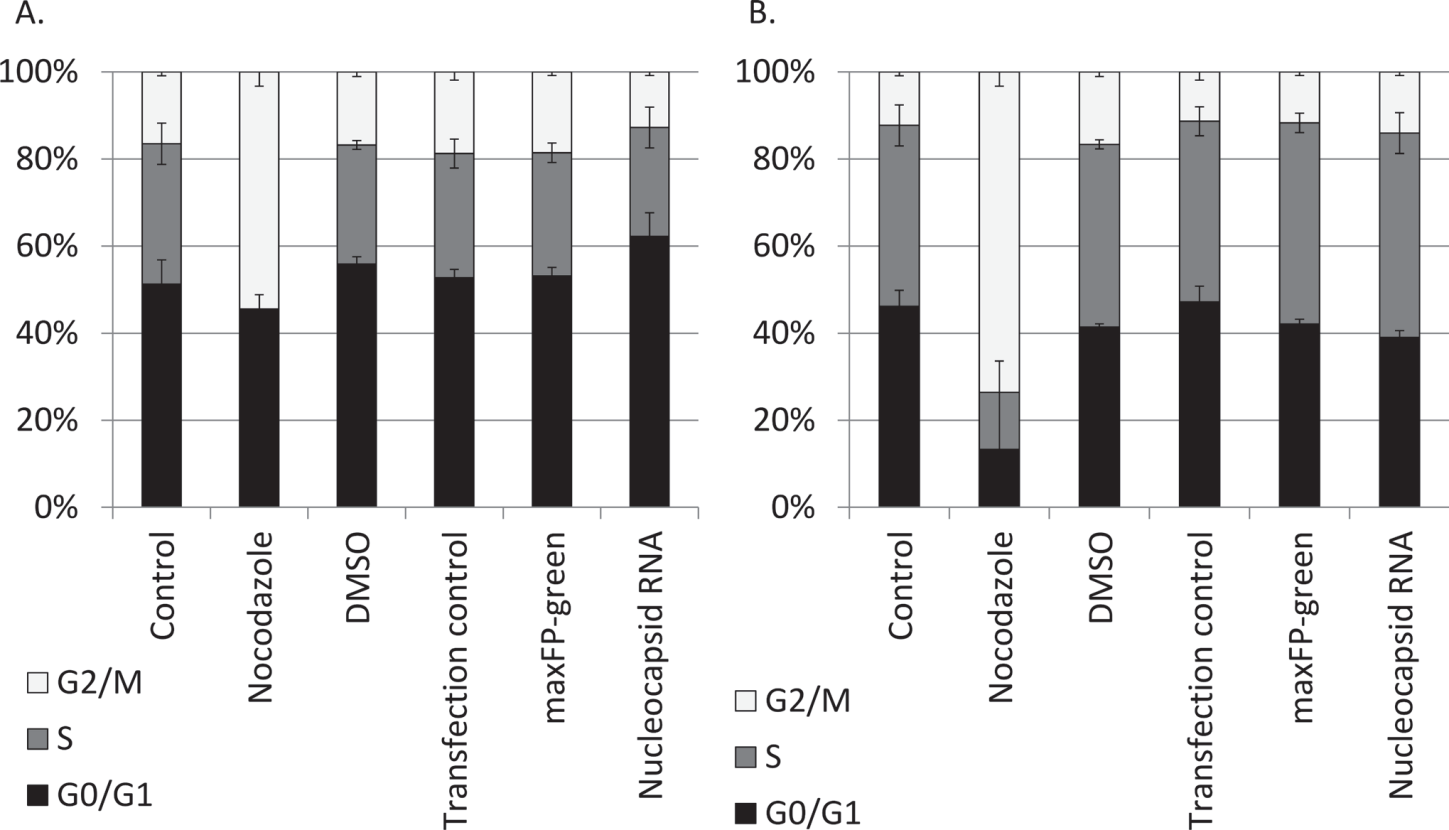
Cell cycle analysis conducted on cells transfected with the NL63-N encoding mRNA or control, maxFP-green encoding mRNA. Analysis was performed using LLC-MK2 cells (**A**.) and 293T cells (**B**.). Nocodazole was used as a positive control, while non-viral mRNA and DMSO were used as negative controls.

To examine whether overexpression of the N protein affects cell cycle progression, we transfected LLC-MK2 and 293T cells with RNA encoding NL63-N. In this particular case, we used RNA transfection because it was more efficient than transfecting cells with DNA vectors (unpublished observations). Only cells expressing NL63-N (as identified by staining with specific antibodies) were used for subsequent analyses. There was no significant difference in cell cycle progression in NL63-N-expressing and non-expressing cells (**Fig. 7**).

## Discussion

Since its discovery, HCoV-NL63 was considered to be the closest relative of HCoV-229E. However, later identification of novel bat coronavirus species revealed several alphacoronaviruses closely clustering with the two human pathogens. Such an observation raised speculations on zoonotic origin of HCoV-NL63 [63,64]. Considering that HCoV-NL63 is rather an atypical alphacoronavirus (e.g., in terms of receptor usage and the predicted protease active site [26,29], it has been suggested that, despite the high similarity between HCoV-229E and HCoV-NL63 [65], these viruses may represent two distinct species that evolved from a common ancestor in bats, and were then introduced into human population *via* two independent zoonotic transmission events [63,64].

The NL63-N is a basic protein comprising of 377 aa. Both the NTD and the complete N protein are unstable, as shown by the broad, irreversible DSC curves and obtained thermal parameters values: T_m_ below 50°C and low ΔH value for complete N protein. In general, ΔH represents the energy amount of non-covalent bonds occurring within native protein. This is consistent with our finding that the N protein rapidly degrades in aqueous solutions (data not shown). Obtained results are consistent with previous reports [55,66,67]. However, the CTD was surprisingly stable; no protein aggregation was observed upon heating and thermal denaturation was fully reversible, moreover judging from *ΔH(T*_*m*_)/ *ΔH*_*vH*_ ratio it undergoes cooperative transition.

Thermodynamic protein stability is defined as the Gibbs energy difference between the denatured and native states but, for some practical purposes, the denaturation temperature, T_m_ (where ΔG = 0 for two-state reversible transition) may be more useful to measure the protein stability. Irreversibility is a common feature of DSC measurements when the large, multidomain proteins are considered. Irreversibility limits the use of a standard equilibrium thermodynamic analysis. For such a process, the denaturation temperature measured in DSC experiment is significantly lower than that corresponding to ΔG = 0. In these cases, the T_m_ (so called operational thermal stability) is kinetically controlled. The simplest model of irreversible unfolding can be described in the terms of kinetic analysis (rate equations) according to general Lumry-Erying scheme:

$$N\overset{K}{↔}U\overset{k}{\to }D$$

where *K* is the equilibrium unfolding constant characterizing reversible unfolding step, *k* is the first order rate constant describing following irreversible step; *N*, *U* and *D* correspond to native, reversibly unfolded and irreversibly denatured monomer of the protein, respectively. Therefore, D represents a modified denatured state existing in a quasi-two-state equilibrium with the native state [68,69]. In fact, for irreversible denaturation the enthalpy and T_m_ become only apparent values since both parameters are dependent on the scan rate. The existence of the exotherm on the downhill part of endotherm DSC peak is the obvious evidence for the presence of association-aggregation processes—the most common reason of the protein unfolding irreversibility.

Lower stability of NTD and the complete N protein in comparison to CTD could be attributed to the distinct structural flexibility of these proteins. In turn, higher flexibility allows NTD to bind the nucleic acids which has obvious biological relevance for NTD and complete N protein function.

One of the major functions of the N protein is to bind viral RNA to form a nucleoprotein, which is then packed into new virions. Therefore, we next examined the ability of NL63-N to bind nucleic acids in an electrophoretic mobility shift assay. Our results confirmed that the NTD efficiently bound RNA and DNA. However, previous studies show that coronaviral N protein has a higher affinity for the viral RNA than non-viral nucleic acids and that only nucleoproteins with coronaviral genomic RNA are efficiently incorporated into new virions [51,70–72]. Observed limited binding of the nucleic acids by the complete N protein (data not shown) hypothetically resulted from interaction between the NTD and CTD. However, rapid N-protein degradation at the conditions of the experiment did not allow us to obtain conclusive answer. This phenomena requires further studies, as we were not able to distinguish at this stage the mechanism of NTD-CTD interaction that limits nucleic acid binding and most likely increases the binding specificity.

Previous studies demonstrated that coronaviral N proteins form dimers, and to lesser extent higher order oligomers [55]. The crystal structures of the SARS–CoV, infectious bronchitis virus (IBV), and mouse hepatitis virus (MHV)-N CTDs revealed that all those domains are characterized by a similar polypeptide fold and are dimeric, strongly suggesting that the dimeric N protein is the unit that functions *in vivo* [56,57]. Furthermore, Tang *et al*. compared the N proteins from SARS-CoV and HCoV-229E [55] demonstrating that they formed oligomers and bound to nucleic acids. Moreover, Lo *et al*. showed that oligomerization of the 229E-N protein is most likely also mediated by the CTD [57]. Here we show that the complete NL63-N protein and the CTD can self-associate to form dimers and higher order oligomers. The role of aforementioned N-N interaction is, however, debatable, as NL63-N strongly binds nucleic acids during formation of the ribonucleocapsid. It is therefore possible that this interaction may be important for stabilizing and shaping the ribonucleocapsid; however, it may also be important for other N-mediated processes [73].

N protein of most coronaviruses is abundantly present in the nucleus of the infected cell [58–61]. Analysis of the NL63-N protein using PSORT II revealed the presence of two NLS (pat4 and pat7), both of which are homologous to those observed in other coronaviral N proteins. For example, the IBV virus carries two very similar putative NLS signals: pat4 RPKK [aa 359–362] and pat7 PKKEKKL [aa 360–366]. One may, therefore, expect that the NL63-N protein would also localize to the nucleus. Surprisingly, no sign of NL63-N localization to the nuclei of infected or transfected cells was detected, suggesting that these NLS motifs are buried within the NL63-N structure, as previously proposed for SARS-CoV [58,74].

To validate that the solely cytoplasmic localization of NL63-N is found not only in established cell lines, we tested a fully differentiated human airway epithelium cultures, mimicking the natural environment of the human airway epithelium; identical results were obtained. It is, however, possible that the lack of nuclear staining observed after natural infection or transfection with the NL63-N-encoding plasmid may be due to poor antibody staining within the nucleus [75]. To address this problem, we used a vector encoding maxFP-Green-N/NL63-N fusion protein, but found no difference in the subcellular localization of the fusion protein and the native protein [60,76,77]. Also, we found that overexpression of maxFP-Green-N/NL63-N in the cells yielded a staining pattern identical to that shown by the native N protein. Presented data show that the NL63-N protein does not localize to the nucleus (or does so in a very limited fashion), similarly to the SARS-CoV N protein and differently than other coronaviral N proteins [60,76].

Nuclear localization of the N protein may be important for several processes, including direct interference with the cell cycle. Dysregulation of the cell cycle is a common strategy used by many DNA and RNA viruses, which enables them to hijack and exploit the host cell machinery for their own benefit. Indeed, the N proteins of many coronaviruses (including SARS-CoV; although the N protein does not localize into the nucleus in this case) inhibit cell cycle progression [36–38,61]. However, we found no difference in the proportion of cells in each phase of cell cycle for NL63-N protein-expressing and non-expressing cells.

In conclusion, we demonstrated here that although NL63-N is largely similar to other coronaviral N proteins, it possesses some unique characteristics: it does not localize to the nucleus of the infected cell and its expression does not appear to affect the cell cycle progression.

## Supporting Information

S1 File Design of the N- and C-terminal domains of HCoV-NL63 N protein expression constructs was based on the sequence alignment with homologous N proteins together with the structural comparative analysis. *Upper panel*: In order to predict polypeptide sequence encompassing the N-terminal domain of N protein from HCoV-NL63 the following sequences of N proteins were aligned: Q6Q1R8 (Uniprot ID), HCoV-NL63; P69596, avian infectious bronchitis virus (IBV, strain Beaudette), sequence referring to the 2bxx crystal structure (Protein Data Bank ID); P69598, IBV (strain Beaudette US), sequence referring to the 2c86 crystal structure; P32923, IBV (strain Gray), sequence referring to the 2gec crystal structure; P03416, murine hepatitis virus (strain A59), sequence referring to the 3hd4 crystal structure; P59595, SARS CoV, sequence referring to the 2ofz crystal structure, P59595, SARS CoV, sequence referring to the 2og3 crystal structure. Sequences referring to residues 1–210 of N protein from HCoV-NL63 are presented as it is enough to reflect the full N-terminal domains of each sequence aligned. Sequences reflecting residues defined by the electron density maps of the crystal structures (2bxx, 2c86, 2gec, 3hd4, 2ofz, 2og3) are colored green. The sequence of HCoV-NL63 N protein predicted to constitute the structurally stable N-terminal domain is colored blue. To predict polypeptide sequence encompassing the C-terminal domain of N protein from HCoV-NL63 the following sequences of N proteins were aligned: Q6Q1R8, HCoV NL63; P69596, avian infectious bronchitis virus (IBV, strain Beaudette), sequence referring to the 2ca1 crystal structure; P32923, IBV (strain Gray), sequence referring to the 2ge7 crystal structure; P32923, IBV (strain Gray), sequence referring to the 2ge8 crystal structure; P59595, human SARS coronavirus, sequence referring to the 2cjr crystal structure; P59595, SARS CoV, sequence referring to the 2jw8 solution structure. Sequences referring to residues 194–377 of N protein from HCoV-NL63 are presented as it is enough to reflect the full C-terminal domains of each sequence aligned. Sequences reflecting residues defined by the electron density maps of the N protein CTD crystal structures (2ca1, 2ge7, 2ge8, 2cjr) and the residues of 2jw8 solution structure are colored violet. The sequence of HCoV-NL63 N protein predicted to constitute the structurally stable C-terminal domain is colored red. *Lower panel*: Superposition of N protein NTD and CTD structures used in the comparative analysis. Multiple molecules composing the asymmetric unit of given structure are colored the same. Multialignment was performed in MUSCLE [78]. Comparative structural analysis was done in SPDBV, Coot and PyMol [47,79]. Figures of the superimposed protein structures were made in PyMol.
(TIF)

S2 File Primary sequence of the NTD and CTD domains of the N-NL63 protein. (TIF)

S3 File Subcellular localization of NL63-N protein in 293T_ACE2^+^ cells infected with HCoV-NL63. Virions were labelled with antibodies specific to the N-NL63 protein (green); DNA was stained with DAPI (blue). Analysis was carried on with confocal microscopy using Leica TCS SP5 II confocal microscope. Voxel size: 31.1 × 31.1 × 167.8 nm, step size: 170 nm, scale bar: 5 μm.
(AVI)

S4 File Subcellular localization of NL63-N protein in LLC-MK2 cells infected with HCoV-NL63. Virions were labelled with antibodies specific to the N-NL63 protein (green); DNA was stained with DAPI (blue). Analysis was carried on with confocal microscopy using Leica TCS SP5 II confocal microscope. Voxel size: 61.7 × 61.7 × 167.8 nm, step size: 170 nm, scale bar: 10 μm.
(AVI)

S5 File Subcellular localization of NL63-N protein in fully differentiated human airway epithelium cultures cells infected with HCoV-NL63. Virions were labelled with antibodies specific to the N-NL63 protein (green); DNA was stained with DAPI (blue). Analysis was carried on with confocal microscopy using Leica TCS SP5 II confocal microscope. Voxel size: 49.9 × 49.9 × 125.9 nm, step size: 130 nm, scale bar: 10 μm.
(AVI)
