# Supplementary figures and images for: The Nucleocapsid Protein of Human Coronavirus NL63
Source: PLoS One. 2015 Feb 20;10(2):e0117833. doi: 10.1371/journal.pone.0117833 (PMC4336326; doi:10.1371/journal.pone.0117833)

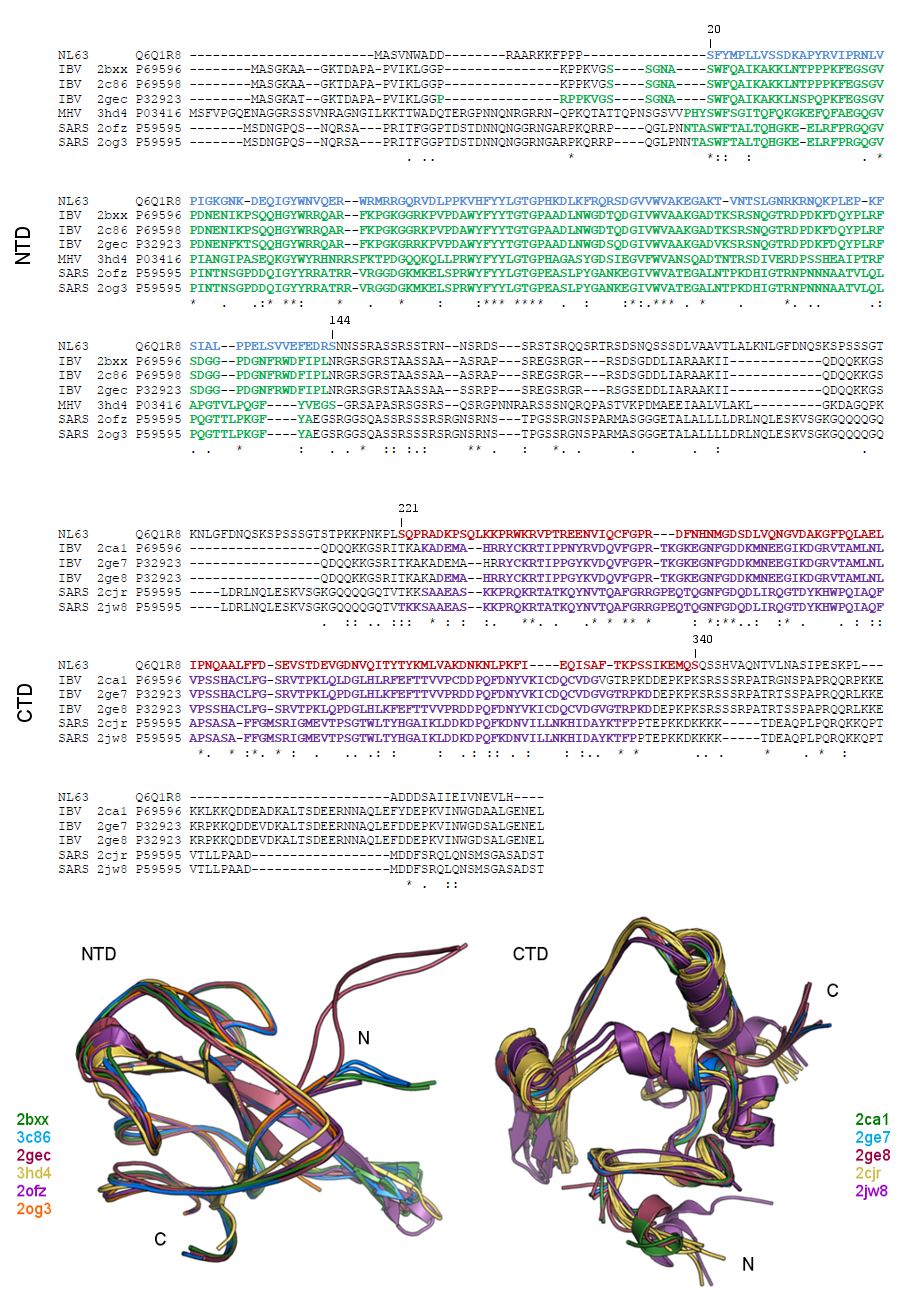

Supplement: S1 File — Upper panel: In order to predict polypeptide sequence encompassing the N-terminal domain of N protein from HCoV-NL63 the following sequences of N proteins were aligned: Q6Q1R8 (Uniprot ID), HCoV-NL63; P69596, avian infectious bronchitis virus (IBV, strain Beaudette), sequence referring to the 2bxx crystal structure (Protein Data Bank ID); P69598, IBV (strain Beaudette US), sequence referring to the 2c86 crystal structure; P32923, IBV (strain Gray), sequence referring to the 2gec crystal structure; P03416, murine hepatitis virus (strain A59), sequence referring to the 3hd4 crystal structure; P59595, SARS CoV, sequence referring to the 2ofz crystal structure, P59595, SARS CoV, sequence referring to the 2og3 crystal structure. Sequences referring to residues 1–210 of N protein from HCoV-NL63 are presented as it is enough to reflect the full N-terminal domains of each sequence aligned. Sequences reflecting residues defined by the electron density maps of the crystal structures (2bxx, 2c86, 2gec, 3hd4, 2ofz, 2og3) are colored green. The sequence of HCoV-NL63 N protein predicted to constitute the structurally stable N-terminal domain is colored blue. To predict polypeptide sequence encompassing the C-terminal domain of N protein from HCoV-NL63 the following sequences of N proteins were aligned: Q6Q1R8, HCoV NL63; P69596, avian infectious bronchitis virus (IBV, strain Beaudette), sequence referring to the 2ca1 crystal structure; P32923, IBV (strain Gray), sequence referring to the 2ge7 crystal structure; P32923, IBV (strain Gray), sequence referring to the 2ge8 crystal structure; P59595, human SARS coronavirus, sequence referring to the 2cjr crystal structure; P59595, SARS CoV, sequence referring to the 2jw8 solution structure. Sequences referring to residues 194–377 of N protein from HCoV-NL63 are presented as it is enough to reflect the full C-terminal domains of each sequence aligned. Sequences reflecting residues defined by the electron density maps of the N protein CT [file pone.0117833.s001.tif]

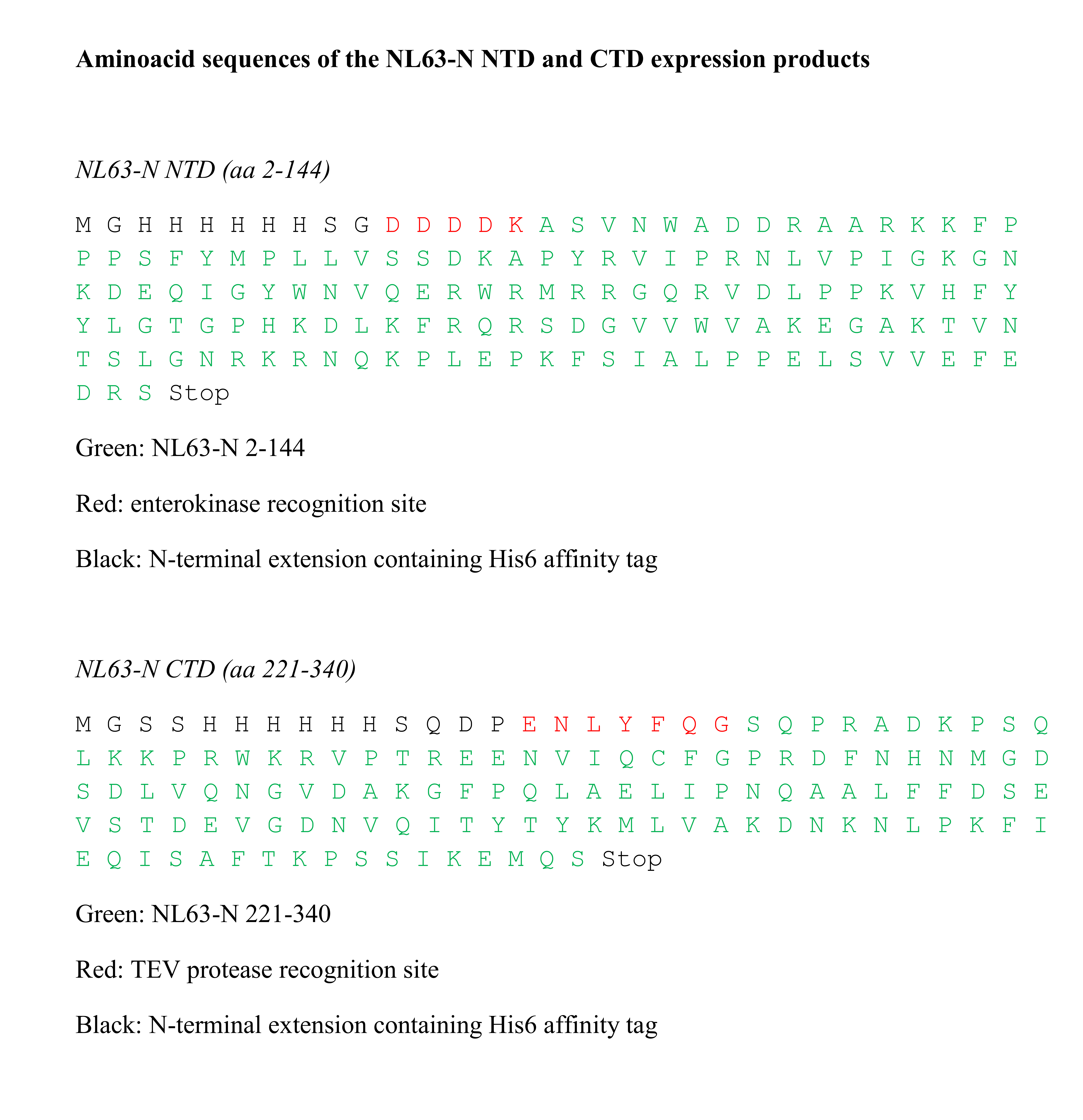

Supplement: S2 File — (TIF) [file pone.0117833.s002.tif]
